# Attention-Enhanced Defensive Distillation Network for Channel Estimation in V2X mm-Wave Secure Communication

**DOI:** 10.3390/s24196464

**Published:** 2024-10-07

**Authors:** Xingyu Qi, Yuanjian Liu, Yingchun Ye

**Affiliations:** 1 College of Electronic and Optical Engineering, Nanjing University of Posts and Telecommunications, Nanjing 210023, China; 2 Office for First-Class Disciplines and High-Level University Construction, Nanjing University of Posts and Telecommunications, Nanjing 210023, China; 3 School of Communication and Information Engineering, Nanjing University of Posts and Telecommunications, Nanjing 210003, China

**Keywords:** mm-wave, V2X, deep learning defensive distillation, attention, secure communication, adversarial attack

## Abstract

Millimeter-wave (mm-wave) technology, crucial for future networks and vehicle-to-everything (V2X) communication in intelligent transportation, offers high data rates and bandwidth but is vulnerable to adversarial attacks, like interference and eavesdropping. It is crucial to protect V2X mm-wave communication from cybersecurity attacks, as traditional security measures often fail to counter sophisticated threats and complex attacks. To tackle these difficulties, the current study introduces an attention-enhanced defensive distillation network (AEDDN) to improve robustness and accuracy in V2X mm-wave communication under adversarial attacks. The AEDDN model combines the transformer algorithm with defensive distillation, leveraging the transformer’s attention mechanism to focus on critical channel features and adapt to complex conditions. This helps mitigate adversarial examples by filtering misleading data. Defensive distillation further strengthens the model by smoothing decision boundaries, making it less sensitive to small perturbations. To evaluate and validate the AEDDN model, this study uses a publicly available dataset called 6g-channel-estimation and a proprietary dataset named MMMC, comparing the simulation results with the convolutional neural network (CNN) model. The findings from the experiments indicate that the AEDDN, especially in the complex V2X mm-wave environment, demonstrates enhanced performance.

## 1. Introduction

V2X is significant for intelligent transportation, facilitating smooth interactions among vehicles (V2V), between vehicles and infrastructure (V2I), with pedestrians (V2P), and across networks (V2N). The evolution of V2X networks has been motivated by the necessity to improve road safety, optimize traffic flow, and enhance the overall driving experience. In future V2X networks, artificial intelligence (AI) and mm-wave technology will be pivotal. Mm-wave technology has emerged as a crucial element in current and future wireless networks thanks to its capability to deliver high data rates and facilitate high-capacity communication. While offering high data rates and a large bandwidth, mm-wave communication is susceptible to security vulnerabilities such as eavesdropping and interference due to its narrow beam and line-of-sight nature [1,2]. Moreover, mm-wave signals are highly sensitive to physical layer attacks, including jamming and spoofing, which can severely impact the reliability of V2X networks [1,2].

Over the last ten years, AI, particularly deep learning (DL), has found extensive applications in millimeter-wave communication. At present, several popular deep learning (DL) models encompass the following: CNNs are effective in capturing spatial features but struggle with temporal dependencies and perform poorly when data are scarce or noisy [3,4]; Recurrent Neural Networks (RNNs) can capture sequential data but suffer from vanishing gradients and are computationally expensive, limiting real-time application [5]; Long Short-Term Memory (LSTM) improves long-term dependency handling but is resource-intensive and prone to overfitting with limited data [6]; and Generative Adversarial Networks (GANs) are helpful for data augmentation but suffer from training instability and are susceptible to adversarial attacks [5,6].

Considering the limitations of mainstream DL models, attention-based models have emerged as a promising solution. Attention mechanisms enable models to concentrate on the most pertinent aspects of input data, enhancing performance in dynamic and intricate settings, like V2X millimeter-wave communication. The attention mechanism, particularly multi-head attention, functions by varying weights to different parts of an input sequence based on relative importance. It does so by projecting the input into query, key, and value vectors, where the query represents the token to be evaluated, the key indicates the context, and the value carries the information to be passed forward. The attention scores, computed as the scaled dot product of queries and keys, are normalized using a softmax function to produce attention weights, which are then used to generate a weighted sum of the values. Multi-head attention extends this mechanism by applying multiple sets of queries, keys, and values in parallel, allowing the model to capture diverse relationships in the data. This enables the model to focus on multiple aspects of the input simultaneously, enhancing its ability to learn complex patterns across various tasks. Unlike traditional models, attention models can efficiently capture spatial and temporal dependencies, providing more accurate channel estimation and enhancing robustness against adversarial attacks.

This study proposes an AEDDN model designed to improve channel estimation accuracy and adversarial robustness in V2X mm-wave communication. By integrating attention mechanisms with defensive distillation, the model focuses on critical features during channel estimation, enhancing its ability to resist adversarial attacks. The primary contributions of this research are outlined below:

1. This research integrates the attention mechanism into mm-wave channel estimation for V2X, enhancing the model’s capacity to concentrate on essential channel characteristics. The attention mechanism reduces noise and irrelevant data by allowing the model to prioritize and assign weights to the most relevant input features. This leads to more accurate channel estimation and better resilience to environmental variations;
2. The attention mechanism is integrated with the defensive distillation method to form a new approach named AEDDN. This method is applied to V2X channel estimation and adversarial attack mitigation, demonstrating superior performance compared to traditional CNN-based methods;
3. The AEDDN method is applied in a complex V2X mm-wave simulation environment to evaluate its performance under realistic conditions. Testing the AEDDN model in this environment demonstrates its robustness, accuracy in channel estimation, and effectiveness in mitigating adversarial attacks.

The rest of this paper is organized as follows: Section 2 reviews the literature pertinent to this study. Section 3 describes the AEDDN method and its efficacy in V2X channel estimation, as well as its role in countering adversarial attacks. Section 4 presents the experimental findings along with the numerical analyses. Lastly, Section 5 concludes this paper.

## 2. Related Work

V2X systems require wireless communication solutions that provide fast data transfer, ultra-low latency, and reliable connections to facilitate real-time data transmission [7]. Due to the sensitive nature of V2X communications, robust security measures are essential to protect against unauthorized access and ensure data integrity [8]. Mm-wave technology enhances V2X communications by offering high bandwidth and low latency, essential for applications like collision avoidance and autonomous driving [9]. Moreover, mm-wave signals provide inherent security benefits with their narrow beamwidth and directionality, reducing the risk of eavesdropping and interference [10]. Despite these advantages, V2X mm-wave channels remain vulnerable to adversarial attacks such as jamming, spoofing, and eavesdropping, which can compromise communication [11]. Traditional security measures like encryption are often insufficient in V2X environments due to the unique challenges of mm-wave communication, including high path loss and physical layer vulnerabilities [12]. Therefore, advanced security solutions are needed to address these challenges.

DL techniques can detect and mitigate various types of attacks by learning from historical data and identifying patterns indicative of malicious behavior [13,14]. Recent studies demonstrate that DL techniques effectively detect and mitigate attacks in wireless communication systems [15,16]. Sim et al. used DL to enhance mm-wave security by detecting jamming and spoofing attacks [17]. At the same time, Yuan et al. applied reinforcement learning to optimize real-time security in V2X communications [18]. However, many existing DL-based solutions are computationally intensive and tailored to specific types of attacks, limiting their generalizability and effectiveness in diverse and dynamic environments like V2X networks [19]. Additionally, these models are often susceptible to adversarial examples, which are carefully crafted inputs designed to deceive DL systems. This underscores the need for more robust DL solutions [5].

Attention mechanisms address the limitations of DL by allowing models to concentrate on the essential elements of the input data, enhancing effectiveness and adaptability in environments like V2X networks [20]. Weighting input features boost robustness against adversarial examples and offer an adaptable framework for complex communication systems [21,22]. Wang et al. applied attention to computer vision, improving object detection by focusing on critical image regions [23]. Choromanski et al. proposed low-rank attention, reducing the computational complexity of large-scale transformers [24]. Lin et al. employed attention to optimize multi-sensor fusion, enhancing the efficiency of sensor networks [25].

Additionally, defensive distillation acts as a strategy to improve the robustness of DL models against threats. This technique involves training a distilled model that exhibits reduced sensitivity to alterations in input, thereby seeking to bolster the model’s capacity to withstand adversarial interference [26,27]. This approach has demonstrated potential across multiple fields by decreasing the model’s sensitivity to particular features and improving its stability [28,29]. In the field of millimeter-wave communication, defensive distillation can enhance the robustness of models against threats, thereby improving the security of V2X communication systems [30,31]. However, the process of defensive distillation within mm-wave channels remains in the initial phases, and further investigation is necessary to improve its efficacy in this field [32,33].

## 3. System Model

### 3.1. The Framework of AEDDN Method

**Figure 1:**
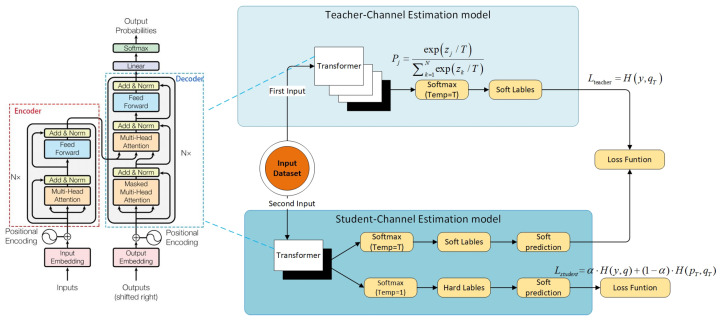
Structure of AEDDN algorithm.

Figure 1 illustrates the general architecture of the AEDDN. In constructing models for teachers and students using the transformer approach, the teacher model—being more complex and larger—is essential in the defensive distillation process. It is trained to learn the features of adversarial samples in V2X communication scenarios and uses a high-temperature softmax to smooth out the effects of input perturbations. Key components include embedding layers for input signal encoding, positional encoding to account for temporal relationships, self-attention mechanisms to capture dependencies, multi-head attention to focus on various input features, and a feed-forward network to improve non-linear representations.

The teacher model is trained using V2X communication data and adversarial samples to produce smooth probability distributions for guiding the student model. The student model, a more streamlined variant of the teacher model, is trained using the knowledge gained from the soft labels provided by the teacher. It is designed to have lower computational complexity while maintaining robustness against adversarial attacks. Both models go through input preprocessing and transformer layer stacking, but the student model uses fewer attention heads or hidden units to reduce computational costs. The student model’s loss function combines hard and soft label loss, balancing accuracy with the ability to draw on the teacher model’s smooth output. In addition, the temperature (T) is raised in the distillation process to produce smoother output distributions. This approach helps mitigate adversarial attacks in V2X mm-wave communication while ensuring computational efficiency.

### 3.2. Adversarial Attacks

Adversarial attacks exploit DL model vulnerabilities by introducing subtle perturbations that cause incorrect predictions, threatening applications like autonomous driving and facial recognition. These attacks are categorized as white box (full model access) and black box (unknown parameters, relying on transferability). Papernot et al. [19] demonstrated that adversarial examples transfer between models, exposing widespread vulnerabilities. Defenses like adversarial training and defensive distillation were developed to enhance robustness.

The following delineates the primary categories of adversarial attacks discussed in this article:

(1) The Fast Gradient Sign Method (FGSM) is a popular and simple attack that modifies the input by utilizing the gradient of the loss function to increase the prediction error of the model. The FGSM formula is as follows:
  (1) $$x_{adv}=x+\epsilon \cdot sign\left(\nabla_{x}J\left(\theta ,x,y\right)\right)$$
  In expression (1), $x_{adv}$ is the adversarial example, *x* is the original input, ϵ controls the perturbation size, $\nabla_{x}J\left(\theta ,x,y\right)$ is the gradient of the loss for *x*, and sign(·) gives the gradient direction.
(2) The Projected Gradient Descent (PGD) is stronger than the FGSM, applying several minor perturbations to generate adversarial examples. After each step, the perturbations are projected onto a predefined ϵ-ball to keep the adversarial example within a set distance from the original input.
  The attack performs gradient ascent iteratively to maximize the model’s loss while keeping the perturbation constrained within an ϵ-ball around the original input. The PGD update rule at step *t* is given by the following:
  (2) $$x_{\mathrm{adv}}^{t+1}=\Pi_{B\left(x,\epsilon \right)}\left(x_{\mathrm{adv}}^{t}+\alpha \cdot sign\left(\nabla_{x}J\left(\theta ,x_{\mathrm{adv}}^{t},y\right)\right)\right)$$
  where $x_{\mathrm{adv}}^{t+1}$ is the adversarial example at step *t*, α is the step size, and $\Pi_{B\left(x,\epsilon \right)}$ is the projection operator that ensures the adversarial example stays within the ϵ-ball centered at the original input *x*.
(3) The Carlini and Wagner (C&W) attack is a highly effective adversarial method that minimizes perturbation while misleading the model. Unlike the FGSM or PGD, it formulates adversarial example generation as an optimization problem aimed at minimizing a particular loss function. The C&W attack minimizes a custom loss function that balances the size of the perturbation and the likelihood of misclassification:
  (3) $$\min_{\delta }\;\|\delta {\|}_{2}+c\cdot f\left(x+\delta \right)$$
  where $\|\delta {\|}_{2}$ is the size of the perturbation, and f(x+δ) ensures the adversarial example is misclassified.

### 3.3. Defense Distillation

Defense Distillation, introduced by Hinton et al. [34], improves neural network robustness by training a ‘student’ model to replicate a ‘teacher’ model’s predictions using softened outputs. Initially, for model compression, it now enhances security and resistance to adversarial attacks. Initially, for model compression, it now enhances security and resistance to adversarial attacks. The initial phase of Defense Distillation consists of training the teacher model through conventional supervised learning. To achieve a smoother output from the teacher during inference, the temperature (*T*) is incorporated into the softmax function. The function is defined as follows:

(4) $$P_{j}=\frac{\exp\left(z_{j}/T\right)}{\sum_{k=1}^{N}\exp\left(z_{k}/T\right)}$$

where $z_{j}$ is the logit (pre-softmax score) for class *j*. When *T* > 1, the output probabilities are softened, spreading the probability mass evenly across all classes.

The teacher model is trained by the traditional cross-entropy loss with the true labels *y*:

(5) $$L_{teacher}=H\left(y,q_{T}\right)$$

where $q_{T}$ is the softmax output at temperature *T*, and $H\left(y,q_{T}\right)$ represents the cross-entropy between the true labels and the teacher’s predictions.

The student model is then trained to imitate the teacher’s smoothed output by utilizing a unified loss function that includes both cross-entropy loss and the distillation loss derived from the teacher’s predictions.

(6) $$L_{student}=\alpha \cdot H\left(y,q\right)+\left(1-\alpha \right)\cdot H\left(p_{T},q_{T}\right)$$

Here, H(y,q) and $H(p_{T})$ are the cross-entropy loss, while α denotes a hyperparameter that regulates the balance between these two loss components.

### 3.4. Transformer Attention Mechanisms

Attention mechanisms were introduced to address limitations in sequence models like RNNs and were first applied to neural machine translation. They allow for models to focus on relevant input parts, handling tokens simultaneously and assigning weights based on importance. This improves performance in tasks with long-range dependencies, such as translation and speech recognition, and helps mitigate issues like vanishing gradients. The transformer [34] is a highly influential attention-based model in DL, using self-attention without recurrent layers. It overcomes RNN limitations by enabling parallel processing, accelerating training, and effectively modeling long-range dependencies.

The self-attention mechanism, which is fundamental to the transformer architecture, enables each token in a sequence to attend to every other token, thereby capturing global relationships. It employs three elements—query *Q*, key *K*, and value *V*—that are obtained from the input through linear transformations:

(7) $$Q=XW^{Q},K=XW^{K},V=XW^{V}$$

The attention score is calculated by performing a dot product between *Q* and *K*, followed by scaling it with the square root of the dimensionality of the key vector, denoted as $d_{k}$, to prevent large values. These computed scores are normalized using the softmax function and then applied to the matrix:

(8) $$\mathrm{Attention}\left(Q,K,V\right)=\mathrm{softmax}\left(\frac{QK^{T}}{\sqrt{d_{k}}}\right)V$$

The transformer uses multi-head attention, dividing the input into multiple heads that compute attention independently, allowing the model to recognize various relationships present in the sequence:

(9) $$\mathrm{MultiHead}\left(Q,K,V\right)=\mathrm{Concat}\left(\mathrm{hea}d_{1},\ldots ,{\mathrm{head}}_{h}\right)W^{O}$$

And each attention head is computed as follows:

(10) $${\mathrm{head}}_{i}=\mathrm{Attention}\left(QW_{i}^{Q},KW_{i}^{K},VW_{i}^{V}\right)$$

Transformers do not have a sequential architecture, so they utilize positional encoding to incorporate information regarding the arrangement of tokens within the sequence. This is achieved by adding sinusoidal positional encodings to the input embeddings:

(11) $$PE_{\left(pos,2i\right)}=\sin\left(\frac{pos}{10000^{2i/d_{\mathrm{moded}}}}\right)$$

(12) $$PE_{\left(pos,2i+1\right)}=\cos\left(\frac{pos}{10000^{2i/d_{\mathrm{moded}}}}\right)$$

Additionally, the transformer applies a feed-forward network to each position after attention, consisting of two linear layers with a ReLU activation in between:

(13) $$FFN\left(x\right)=\max\left(0,xW_{1}+b_{1}\right)W_{2}+b_{2}$$

Residual connections and layer normalization are applied after both the self-attention and feed-forward sublayers to improve gradient flow and stable training:

(14) Output=LayerNorm(x+sublayer(x))

## 4. Experiments

### 4.1. Dataset Description

This study utilized two datasets: the public dataset called the 6g-channel-estimation dataset [35] and the MMMC, a private dataset.

The 6g-channel-estimation dataset is generated with the MATLAB 5G Toolbox. This dataset comprises 256 training sets, each containing 612 subcarriers, 14 OFDM symbols, and a single antenna, resulting in 8568 data points. A CNN-based Defense Distillation method has been created by the dataset developer for channel estimation and the mitigation of adversarial attacks. The 6g-channel-estimation dataset is primarily utilized to evaluate the performance disparity between the AEDDN and CNN-based models.

**Figure 2:**
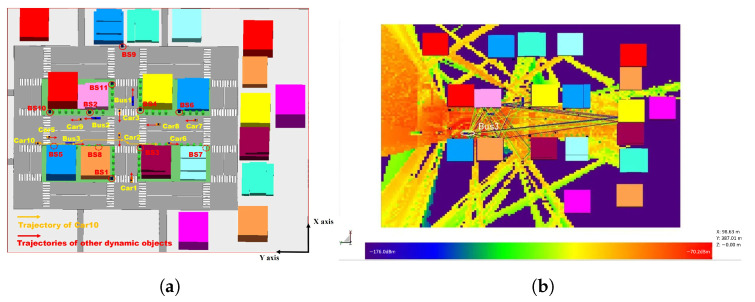
The scenario in the WI simulation platform. (**a**) Specific trajectories of vehicles. (**b**) Heat maps and propagation paths.

The MMMC dataset is a large-scale mm-wave wireless communication dataset for complex high-speed V2X scenarios, simulated using commercial software Wireless InSite (WI), which offers three-dimensional ray-tracing, rapid ray-based techniques, and empirical models tailored for the assessment of localized radio wave propagation and wireless communication systems. The MMMC dataset includes 1500 snapshots of vehicular network wireless communication channel information, covering V2V and V2I communication links. This scene is a complex, high-dynamic urban intersection with 11 base stations, 9 cars, 3 buses, and typical buildings in urban transportation networks. All the base stations and vehicles are both transmitters and receivers. Based on the ray-tracing method, buildings and vehicles can cause reflection, diffraction, and transmission of mm-wave propagation. Figure 2 shows a simulated planar representation of the urban roads and vehicle movements, displaying the positions of the base stations and vehicles and marking the movement directions of all the vehicles. Table 1 provides a summary of the more specific parameters.

### 4.2. Experimental Setting

Our experimental configuration included an Intel(R) Core(TM) i7-7700HQ processor operating at 2.8 GHz, equipped with 32 GB of RAM and an NVIDIA RTX4090 graphics card. The software environment comprised CUDA version 11.8 and Pytorch version 2.2.0, while Python 3.8 was utilized as the programming language for our implementation.

### 4.3. Experimental Results for 6g-Channel-Estimation Dataset

The MSE (Mean Squared Error) and attack success ratio (ASR) are utilized to assess and compare models based on the AEDDN and CNN. The MSE is a regression loss function that squares discrepancies between the forecasted value ${\hat{x}}_{i}$ and actual value $x_{i}$, sums them, and divides them by the number of instances *j*, emphasizing larger errors. The formula for the MSE is as follows:

(15) $$\mathrm{MSE}=\frac{1}{j}\underset{i=1}{\sum^{j}}\;{\left(x_{i}-{\hat{x}}_{i}\right)}^{2}$$

The ASR measures the ratio of successful attacks to total attacks, indicating a model’s vulnerability to adversarial manipulation. The formula for the ASR is as follows:

(16) $$\mathrm{ASR}=\frac{\mathrm{Number}\;of\;successful\;attacks}{\mathrm{Total}\;number\;of\;attacks}=\frac{n_{success}}{n_{total}}$$

where $n_{success}$ is the successful attack number, $n_{total}$ is the total attack number.

**Figure 3:**
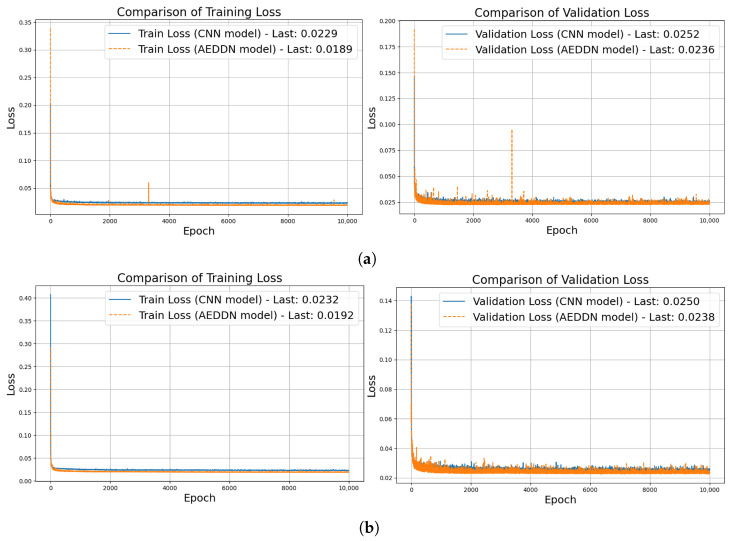
Training history for CNN-based and AEDDN models through 6g-channel-estimation dataset. (**a**) Teacher model training. (**b**) Student model training.

The training history for both the teacher and student models on the 6g-channel-estimation dataset is illustrated in Figure 3. The overall trend shows that the AEDDN enhances the performance of the channel estimation model, yielding superior outcomes in mitigating attacks when contrasted with the CNN-based model.

**Figure 4:**
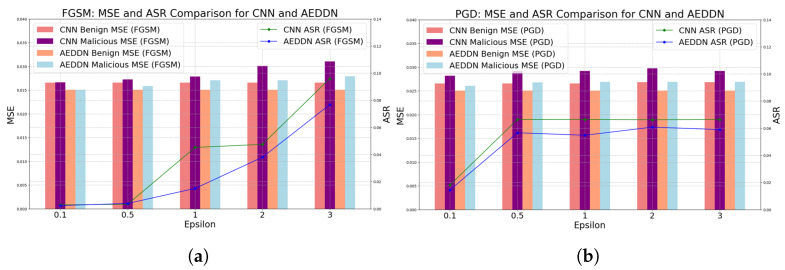
Comparative performance of CNN and AEDDN models under adversarial attacks. (**a**) MSE and ASR comparison under FGSM attack. (**b**) MSE and ASR comparison under PGD attack.

The comparison between the CNN-based and the AEDDN model in facing different adversarial attacks (FGSM, PGD, and C&W) is analyzed in Table 2 and Figure 4. In the FGSM attack, the CNN and AEDDN models show a varying MSE and ASR across ϵ values. For benign inputs, the CNN-based model maintains a stable MSE of 0.026561. In contrast, the AEDDN model performs slightly better with a lower MSE of 0.025066 due to their architectural differences, specifically the use of attention mechanisms in the AEDDN and variations in feature extraction, training processes, and optimization. These factors lead to distinct handling of the same benign inputs despite using the same dataset. For malicious inputs, the CNN’s MSE increases from 0.026623 at ϵ = 0.1 to 0.031010 at ϵ = 3, while the AEDDN exhibits more controlled growth, from 0.025068 to 0.027909. The ASR for the CNN-based model rises sharply from 0.002813 to 0.095989, whereas the AEDDN model shows stronger resistance, with the ASR increasing from 0.002355 to 0.076791. In the PGD attack, similar trends appear. The CNN’s MSE remains stable at around 0.026858 for benign inputs, while the AEDDN maintains lower values between 0.025073 and 0.025084. For malicious inputs, the CNN’s MSE rises from 0.028190 to 0.029185, while the AEDDN stays more stable, between 0.026072 and 0.026882. The CNN’s ASR remains high at around 0.066, while the AEDDN’s ASR is significantly lower, increasing from 0.014447 to 0.058987, demonstrating the AEDDN’s superior robustness against PGD attacks.

The results demonstrate that the AEDDN model consistently has a lower MSE than the CNN model for both benign and malicious inputs, indicating better performance and stronger robustness against adversarial attacks. The ASR values indicate that, compared to the CNN-based model, the AEDDN model demonstrates a reduced susceptibility to adversarial attacks across all scenarios. In contrast, the AEDDN model demonstrates a lower ASR, particularly in FGSM attacks, indicating better defense and improved security. Additionally, as the attack intensity (ϵ) increases, both the CNN-based and AEDDN models see a rising MSE and ASR in FGSM attacks, but the AEDDN shows smaller increases, indicating better robustness. In the PGD attacks, the AEDDN model has a lower MSE and significantly reduced ASR, proving its effectiveness against iterative attacks. For the C&W attacks, the AEDDN model outperforms the CNN in both the MSE and ASR, demonstrating superior defense against complex adversarial attacks.

### 4.4. Experimental Results for MMMC Dataset

**Figure 5:**
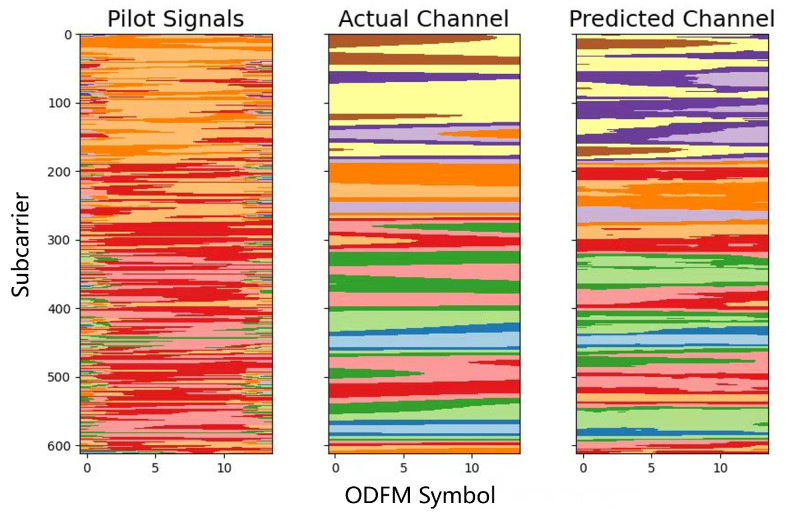
Comparison of pilot signals, actual channel, and predicted channel using AEDDN model for MMMC dataset.

Figure 5 demonstrates the AEDDN model’s effectiveness in reconstructing the channel, consisting of the pilot signals, the actual channel, and the predicted channel. The pilot signals, randomly distributed, help estimate the channel, while the actual channel reflects the true wireless environment. The predicted channel closely matches the actual channel, showing the model’s accuracy. The comparison highlights the AEDDN model’s robustness in handling complex channel conditions, effectively capturing key features through attention mechanisms and defensive distillation.

**Figure 6:**
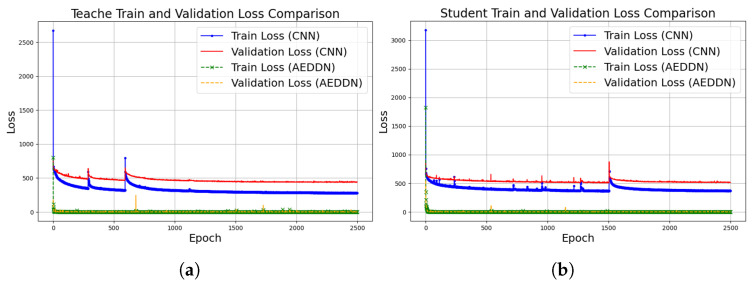
Loss value comparison between CNN-based and AEDDN models through MMMC dataset. (**a**) Loss value from teacher training and validation. (**b**) Loss value from student training and validation.

Figure 6 compares the training and validation loss of CNN-based and AEDDN models on V2X mm-wave communication data. For the teacher model, CNN’s initial training loss reaches as high as 3000, and while it decreases over time, the loss remains high with significant fluctuations, particularly in the validation curve. In contrast, the AEDDN’s loss quickly stabilizes below 500, with a reduction of over 80% in both training and validation losses compared to the CNN-based model, demonstrating better robustness and generalization. For the student model, the CNN shows similar behavior, with initial losses around 2500 and ongoing instability, while the AEDDN maintains much lower and stable loss values throughout the training process. The AEDDN’s consistent performance across both teacher and student models highlights its superior ability to handle adversarial attacks and complex communication environments, providing significantly improved stability and resistance to overfitting.

## 5. Conclusions

This study presents an innovative attention-enhanced defensive distillation network (AEDDN) to enhance the robustness and accuracy of channel estimation in V2X mm-wave communication systems under adversarial attacks. The network’s capacity to withstand adversarial disturbances is bolstered by integrating defensive distillation with attention mechanisms. Through extensive simulation and dataset validation, the AEDDN model demonstrated superior performance over traditional CNN-based methods, particularly in handling attacks, like the FGSM, PGD, and C&W. The results confirm the effectiveness of the proposed solution in securing V2X mm-wave communication systems, offering insights into future research directions for enhanced security measures.

Nonetheless, our current model has not yet succeeded in reducing the training and testing time. Future endeavors will focus more on lightweight models and improving computational efficiency to ensure that our findings can be effectively applied to practical V2X mm-wave communication scenarios.

## Figures and Tables

**Table 1:** Simulation parameters.

Parameter	Value
Carrier frequency	28 GHz
Types of antennas	Half-wave dipole antenna
Bandwidth	2 GHZ
Transmitting power	10 dBm
Noise power	−6.99 dBm
Reflections	4
Diffractions	1
Transmissions	0
Communication link	V2V and V2I

**Table 2:** Numerical comparison between AEDDN and CNN for 6g-channel-estimation dataset.

Attack	ϵ	MSE (CNN)		ASR (CNN)	MSE (AEDDN)		ASR (AEDDN)
		Benign Input	Malicious Input		Benign Input	Malicious Input	
	0.1	0.026561	0.026623	0.002813	0.025066	0.025068	0.002355
	0.5	0.026561	0.027244	0.003349	0.025066	0.025835	0.003879
FGSM	1	0.026561	0.027863	0.045198	0.025066	0.027095	0.014980
	2	0.026564	0.030106	0.047475	0.025066	0.027096	0.037980
	3	0.026561	0.031010	0.095989	0.025066	0.027909	0.076791
	0.1	0.026589	0.028190	0.018059	0.025073	0.026072	0.014447
	0.5	0.026588	0.029177	0.066410	0.025072	0.026795	0.056643
PGD	1	0.026588	0.029177	0.066473	0.025072	0.026898	0.054774
	2	0.026861	0.029729	0.066182	0.025084	0.026883	0.060767
	3	0.026862	0.029185	0.066456	0.025076	0.026882	0.058987
C&W	-	0.026263	0.027408	0.027818	0.025084	0.026154	0.014550

## Data Availability

The data are contained within the article.
